# In Vitro Evaluation of the Phytopharmacological Potential of *Sargassum incisifolium* for the Treatment of Inflammatory Bowel Diseases

**DOI:** 10.3390/medicines6020049

**Published:** 2019-04-06

**Authors:** Mutenta N. Nyambe, Trevor C. Koekemoer, Maryna van de Venter, Eleonora D. Goosen, Denzil R. Beukes

**Affiliations:** 1 Department of Biochemistry and Microbiology, P.O. Box 7700, Nelson Mandela University, Port Elizabeth 6031, South Africa; mutentanyambe@gmail.com (M.N.N.); trevor.koekemoer@mandela.ac.za (T.C.K.); 2 Faculty of Pharmacy, Division of Pharmaceutical Chemistry, P.O. Box 94, Rhodes University, Grahamstown 6140, South Africa; l.goosen@ru.ac.za; 3 School of Pharmacy, Private Bag X17, University of the Western Cape, Bellville 7535, South Africa; dbeukes@uwc.ac.za

**Keywords:** PPAR-γ, sargahydroquinoic acid, sarganaphthoquinoic acid, sargachromenoic acid, inflammation, bowel diseases

## Abstract

**Background:** Comprised of Crohn’s disease and ulcerative colitis, inflammatory bowel diseases (IBD) are characterized by chronic inflammation of the gastro-intestinal tract, which often results in severe damage to the intestinal mucosa. This study investigated metabolites from the South African endemic alga, *Sargassum incisifolium*, as potential treatments for IBD. Phytochemical evaluation of *S. incisifolium* yielded prenylated toluhydroquinones and toluquinones, from which semi-synthetic analogs were derived, and a carotenoid metabolite. The bioactivities of *S. incisifolium* fractions, natural products, and semi-synthetic derivatives were evaluated using various in vitro assays. **Methods:** Sargahydroquinoic acid isolated from *S. incisifolium* was converted to several structural derivatives by semi-synthetic modification. Potential modulation of IBD by *S. incisifolium* crude fractions, natural compounds, and sargahydroquinoic acid analogs was evaluated through in vitro anti-inflammatory activity, anti-oxidant activity, cytotoxicity against HT-29 and Caco-2 colorectal cancer cells, and PPAR-γ activation. **Results:** Sargahydroquinoic acid acts on various therapeutic targets relevant to IBD treatment. **Conclusions:** Conversion of sargahydroquinoic acid to sarganaphthoquinoic acid increases peroxisome proliferator activated receptor gamma (PPAR-γ) activity, compromises anti-oxidant activity, and has no effect on cytotoxicity against the tested cell lines.

## 1. Introduction

The incidence of the two major types of inflammatory bowel diseases (IBDs), Crohn’s disease (CD) and ulcerative colitis (UC), has become a global health challenge. A systematic review of studies reporting the prevalence and incidence of IBDs, performed by Ng et al., revealed that while the incidence has stabilised in the westernised world, it has steadily been increasing in developing countries over the past decade or two [1]. CD and UC are characterised by chronic inflammation of the intestine with many associated symptoms, complications, and an increased risk for colorectal cancer [2]. Conventional treatment is aimed at reducing intestinal inflammation and modulating the immune system. The most commonly used treatments are aminosalicylate anti-inflammatories (5-ASA, sulfasalazine, mesalamine and derivatives), corticosteroids (prednisone, prednisolone, budesonide, budesonide MMX), immunosuppressives (thiopurines, methotrexate) and TNF antagonists (infliximab, adalimumab, certolizumab pegol, golimumab). More recent developments include integrin antagonists to inhibit T cell adhesion and antagonists of the pro-inflammatory interleukins IL-12 and -23 [2]. None of these medications come without problems such as safety, efficacy, or cost implications and the search for new alternatives continues [2,3].

Oxidative stress signalling has been implicated in the pathogenesis and progression of IBD [4]. Although its exact role and mechanism is not fully understood, it is accepted that oxidative stress plays a role in the initiation and development of the disease and is not merely a result of chronic inflammation in the gut. Antioxidants may therefore have potential therapeutic effects especially if administered in combination with conventional therapies [4].

The nuclear receptor PPAR-γ, well known for its role in adipocyte differentiation, has also been identified as a potential therapeutic target for IBD [5,6]. It plays a role in regulation of inflammation in the intestine, where it is expressed at high levels in epithelial cells and at lower levels in macrophages and lymphocytes [7]. Peroxisome proliferator-activated receptor gamma (PPAR-γ) agonists inhibit the inflammatory response in intestinal epithelial cells [4,5] and macrophages [8]. Activation of PPAR-γ also slows down the proliferation of colon cancer cells [9] and protects against the development of colorectal cancer [10].

Secondary metabolites from natural products have been an important source of lead compounds for drug development. Advances in chemical techniques and functional, as well as phenotypic, bioassays have led to a revived interest in this field [11,12]. The multi-target nature of pleiotropic natural products holds many advantages in the treatment of complex diseases [13].

The brown seaweed *Sargassum incisifolium* is found in South Africa (from the Western Cape through the Eastern Cape and KwaZulu-Natal), southern Mozambique, and south-east Madagascar [14]. An aqueous extract of this species was shown to exhibit no antimicrobial activity on its own but surprisingly enhanced the antimicrobial potential of silver nanoparticles [15]. The same authors have reported a high polyphenol content of 150 µg/mg for the aqueous extract and high antioxidant activity, with a total reducing power of 75 ascorbic acid equivalents (AAE), measured in µg/mg of dried extract. Partitioning of the aqueous extract with organic solvent increased the polyphenol content to 235 µg/mg and the reducing power to 95 µg/mL. Although IC_50_ values were not reported by the authors, the extract and organic partition were non-toxic to MCF-7 cells at 100 µg/mL, while reducing HT-29 and MCF-12a cell viability to between 45% and 70% [15].

**Figure 1:**
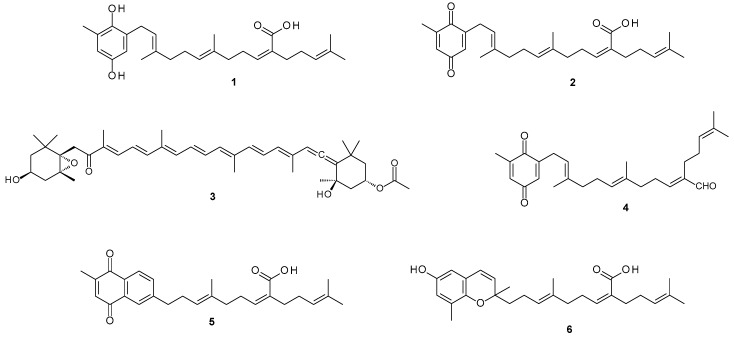
*Sargassum incisifolium* metabolites sargahydroquinoic acid (**1**), sargaquinoic acid (**2**), fucoxanthin (**3**) and sargaquinal (**4**), and semi-synthetic derivatives sarganaphthoquinoic acid (**5**) and sargachromenoic acid (**6**).

This study investigated the potential of metabolites from the South African endemic alga *Sargassum incisifolium* (Figure S1) in the treatment of inflammatory bowel diseases (IBD). Phytochemical evaluation of *Sargassum incisifolium* yielded known compounds consisting of prenylated metabolites and a carotenoid. The isolated natural compounds were sargahydroquinoic acid (SHQA, **1**), sargaquinoic acid (SQA, **2**), fucoxanthin (**3**), and sargaquinal (**4**). Since SQA (**2**) was isolated in minute quantities, it was further semi-synthesized from SHQA (**1**) (65.1% yield). Sarganaphthoquinoic acid (SNQA, **5**) and sargachromenoic acid (SCA, **6**) were semi-synthesized from sargaquinoic acid (**2**) and sargahydroquinoic acid (SHQA, **1**), respectively (Figure 1). The bioactivities of *S. incisifolium* fractions, compounds, and semi-synthetic derivatives were evaluated as potential modulators of inflammatory bowel diseases using various in vitro assays.

## 2. Materials and Methods

### 2.1. Reagents

Culture mediums were sourced from Sigma Aldrich® (Johannesburg, South Africa) and Hyclone^®^ (Thermo Fisher, Logan, UT, USA) while Fetal Bovine Serum (FBS) was obtained from LONZA^®^ (Basel, Switzerland). Chang Liver cells (HeLa derivative) were purchased from Highveld Biologicals, Johannesburg, South Africa and HT29 and Caco2 colorectal carcinoma cell lines from the American Type Culture Collection (Manassas, VA, USA). The EC_50_ values of the test compounds were calculated from a minimum 5-point dose-response curve using a GraphPad Prism 4 software package (GraphPad, San Diego, CA, USA). Liquid chromatography utilised HPLC grade solvents supplied by Lichrosolv^®^ (Merck, Germany). NMR experiments were obtained on a Bruker Avance 400 MHz NMR spectrometer (Bruker Corporation, Billerica, MA, USA) using standard pulse sequences. All HPLC solvents were filtered through a 0.45 μm filter before use. Normal phase HPLC was performed using a Spectra-Physics IsoChrom pump (Spectra-Physics, Santa Clara, CA, USA), a Whatman^®^ Partisil 10 (9.5 mm × 500 mm) semi-preparative column (GE healthcare, Chicago, IL, USA) and a Waters 410 differential refractometer (Waters Corporation, Milford, MA, USA) attached to a 100 mV full scale Rikadenki chart recorder (Rikadenki Electronics GmbH, Freiburg im Breisgau, Germany).

### 2.2. Algal Material

The algal specimen of *S. incisifolium* (collection voucher NDK101124) was collected from Noordhoek, near Port Elizabeth, on the southeast coast of South Africa on 24 November 2010. A specimen (Figure S1) is kept in the seaweed collection at the School of Pharmacy, University of the Western Cape. The algal specimen was transported to the laboratory on ice where it was immediately frozen and stored until the time of extraction. For purposes of identification and authentication, the algal material was morphologically compared with previous voucher specimens of *S. incisifolium*. A voucher specimen (NDK06-5) is kept at the Division of Pharmaceutical Chemistry, Rhodes University, Makhanda, South Africa.

### 2.3. Extraction and Isolation of Bioactive Metabolites

The algal extraction procedure was consistent with previously reported methods [16]. The frozen alga (NDK101124) was allowed to defrost under running distilled water. The defrosted alga was then soaked in MeOH for 1 h, after which the MeOH was decanted and the retained algae heated at 40 °C for 30 min in CH_2_Cl_2_/MeOH (2:1, 150 mL × 3). MeOH and CH_2_Cl_2_/MeOH (2:1) mixtures were pooled and sufficient water added to allow for the separation of the CH_2_Cl_2_ and the MeOH/H_2_O phases. The CH_2_Cl_2_ phase was then collected and dried in vacuo to yield the desired crude extract (12.4 g). A portion of the crude extract (0.95 g) was applied to a silica gel column (10 g) and the column eluted using a series of solvents (50 mL each) of increasing polarity. This yielded the following fractions: **Fr A** (*n*-hexane-EtOAc, 10:0, 17.2 mg), **Fr B** (*n*-hexane-EtOAc, 9:1, 20.7 mg), **Fr C** (*n*-hexane-EtOAc, 8:2, 143.1 mg), **Fr D** (*n*-hexane-EtOAc, 7:3, 284.5 mg), **Fr E** (*n*-hexane-EtOAc, 6:4, 32.6 mg), **Fr F** (*n*-hexane-EtOAc, 4:6, 35.5 mg), **Fr G** (*n*-hexane-EtOAc, 2:8, 6.6 mg), **Fr H** (EtOAc, 2.5 mg), and **Fr I** (MeOH-EtOAc, 1:1, 207.7 mg). **Fr D** contained pure sargahydroquinoic acid (SHQA, **1**, 284.5 mg, 30% extracted yield). Normal phase HPLC of **Fr B** (20.7 mg) using *n*-hexane/EtOAc (9:1) yielded sargaquinal (**4**, 3.0 mg, 15.4% dry weight). **Fr F** contained pure fucoxanthin (**3**, 35.5 mg, 3.74% extracted yield). The structures for compounds **1**, **3**, and **4** were confirmed by spectroscopic methods consistent with previously reported data [17,18]. A summary of the isolation process (Scheme S1) as well as the NMR spectra for compounds **1** (Figures S2 and S3), **4** (Figures S4 and S5) and **3** (Figures S6 and S7) are provided in the Supplementary Materials.

### 2.4. Semi-Synthetic Derivatization of Sargahydroquinoic Acid (**1**) Analogs

#### 2.4.1. Oxidation of Sargahydroquinonic Acid (**1**) to Sargaquinoic Acid (**2**)

As previously reported [19].

#### 2.4.2. Conversion of Sargahydroquinoic Acid (**1**) to Sarganaphthoquinoic Acid (**5**)

As previously reported [19].

#### 2.4.3. Conversion of Sargaquinoic Acid (**2**) to Sargachromenoic Acid (**6**)

As previously reported [19]. The ^1^H NMR spectra for compounds **2**, **5** and **6** (Figure S2) and a summary of their derivatization (Scheme S2) are provided in the Supplementary Materials.

### 2.5. Anti-Inflammatory Assay

The murine peritoneal macrophage cells (RAW267.4) were cultured in DMEM containing 10 % FCS. Cells were seeded into 96 well plates at a density of 8 × 10^4^ cells/well and allowed to attach overnight. The cells were then treated with 1 μg/mL of bacterial lipopolysaccharide (LPS) (SIGMA^®^) and two concentrations of the test sample (12.5 and 25 μg/mL) for 18 h. To measure nitrate levels, 50 μL of the spent culture medium was removed and added to an equal volume of Griess reagent (SIGMA^®^). The absorbance was measured at 540 nm using a microplate reader and the nitrate concentrations were calculated by comparison with the absorbance to sodium nitrate standard solutions. Aminogaunidine (Sigma^®^) was used as positive control to demonstrate the inhibition of nitrate production. Cell viability was simultaneously measured using the standard MTT assay.

### 2.6. 2,2-diphenyl-1-picrylhydrazyl (DPPH) Radical Scavenging Assay

Test samples were diluted in EtOH/H_2_O (1:1) from 10 mg/100 µL stocks prepared in DMSO. A total of 5 μL of each sample was placed into each well of a 96-well plate, followed by the addition of 120 μL of Tris-HCl buffer (50 mM, pH7.4) and 120 μL of freshly prepared DPPH solution (0.1 mM in EtOH). The plate was incubated for 20 min at room temperature, with the absorbance read at 513 nm. The percentage of DPPH radical scavenging was calculated as ((A − B/A) × 100) where A represents the absorbance in the absence of test samples and B represents the absorbance in the presence of test samples. Ascorbic acid was used as a positive control (EC_50_ = 24.07 μg/mL).

### 2.7. 3-(4,5-dimethylthiazol-2-yl)-2,5-diphenyltetrazolium Bromide (MTT) Cytotoxicity Assay

HT-29 and Caco-2 cells were seeded into 96-well culture plates (TTP) at 5 000 cells/well in DMEM supplemented with 10% fetal bovine serum (FBS) and left for 24 h. Algal extracts were added and the cells incubated for a further 48 h, after which the medium was replaced with 200 μL MTT (Sigma^®^) (0.5 mg/mL in DMEM). After 3 h of incubation at 37 °C, the MTT was removed and the purple formazan product dissolved in 200 μL DMSO.

HeLa derivative cells were seeded into 96-well culture plates (TTP) at 10,000 cells/well in EMEM supplemented with 10% fetal bovine serum (FBS) and left for 24 h. Algal extracts and compounds were added and the cells incubated for a further 48 h after which the medium was replaced with 200 μL of MTT (Sigma^®^) (0.5 mg/mL in EMEM). After a further 2 h of incubation at 37 °C, the MTT was removed and the purple formazan product dissolved in 200 μL of DMSO.

Absorbance was measured at 560 nm using a multiwell scanning spectrophotometer (Multiscan MS, Labsystems). All incubation steps were carried out in a 37 °C humidified incubator with 5% CO_2_. IC_50_ and EC_50_ values were calculated from a minimum 5-point dose-response curves using the GraphPad Prism 4 software package.

### 2.8. 3T3-L1 Preadipocyte Differentiation Assay

Prior to the induction of differentiation, 3T3-L1 cells were routinely maintained in DMEM containing newborn calf serum. Cells were seeded at a density of 3000 cells/well into 96-well plates and allowed to reach 100% confluence. Two days post-confluence, the cells were treated for a further two days with DMEM medium, now supplemented with FBS (to induce mitotic clonal expansion) and the indicated concentrations of test compounds or the control substances rosiglitazone and troglitazone (1 μM, final concentration). Cells were then cultured for an additional 7 days in normal culture medium (DMEM, 10% FBS with inducers) and the medium replaced every two to three days. Triglyceride accumulation, a marker for adipocyte differentiation, was measured by Oil red-O staining. The Oil Red-O stained lipids were extracted in isopropanol and measured at 510 nm. The sample results were then compared to controls using a two-tailed Student’s t-test assuming equal variances.

## 3. Results

### 3.1. Anti-Inflammatory Potential of S. incisifolium

**Figure 2:**
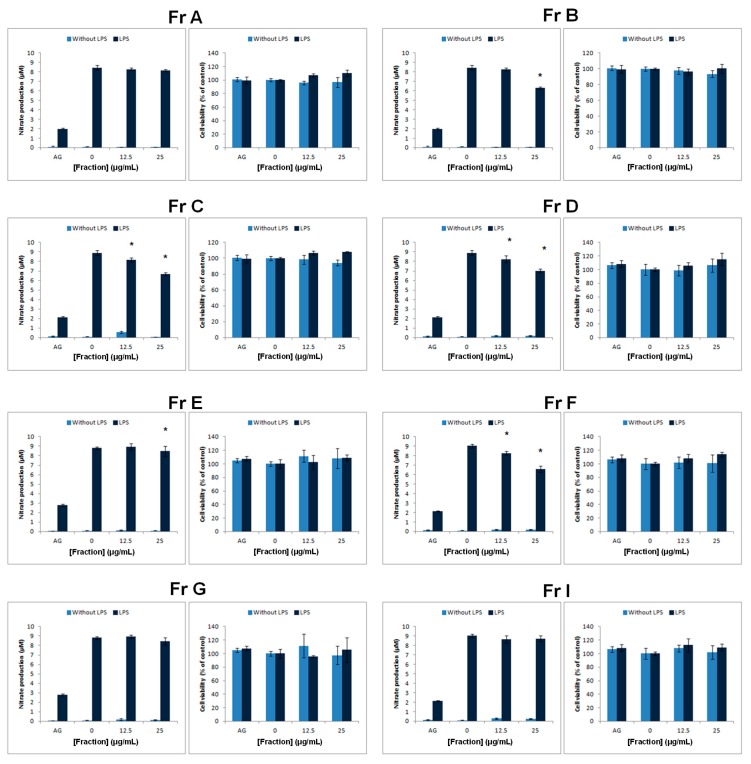
Nitrate production in LPS activated and naïve RAW 264.7 macrophages treated with *S. incisifolium* fractions **Fr A**–**I**. Aminogaunidine (AG) was used as positive control. Data represents the mean ± SD (n = 4). Significant (*p* < 0.05) reductions in the levels of nitrate are indicated as (*).

*S. incisifolium* fractions were evaluated for anti-inflammatory activity. Fractions **Fr C**, **Fr D** (SHQA, **1**), and **Fr F** (fucoxanthin, **3**) produced a significant decrease in LPS-stimulated nitrate production at both test concentrations, with **Fr C** being relatively less potent by only having a significant effect at the highest test concentration (Figure 2). SHQA (**1**) significantly attenuated nitrate production, indicating that this compound may also be considered to possess anti-inflammatory properties. Under the conditions of the anti-inflammatory assay there was no evidence for cytotoxicity toward the RAW 267.4 cells and, thus, it can be assumed that the inhibition of nitrate production was not due to differences in the relative cytotoxicity. In naïve cells, i.e., in the absence of LPS, no response was induced by any of the samples.

### 3.2. Antioxidant Activity of S. incisifolium Fractions, Metabolites, and Derivatives

*S. incisifolium* crude fractions were evaluated for DPPH radical scavenging activity using ascorbic acid as the standard. **Fr C** (EC_50_ = 19.48 µg/mL), **Fr D** (SHQA, **1**) (EC_50_ = 4.01 µg/mL), and **Fr E** (EC_50_ = 3.32 µg/mL) exhibited strong DPPH radical scavenging activity more potent than ascorbic acid (EC_50_ = 24.07 µg/mL) (Table 1). There should be background fucoxanthin absorbance in **Fr F**, which would interfere with the DPPH quantification and, as such, it was not possible to reliably determine its antioxidant activity under these experimental conditions. However, extensive research has already been undertaken to show, among many others, the anti-oxidant, anti-inflammatory and anticancer activity of fucoxanthin (**3**) [20]. SCA (**6**, EC_50_ = 6.99 µg/mL) and SHQA (**1**, EC_50_ = 4.01 µg/mL) exhibited stronger DPPH radical scavenging activity than ascorbic acid. SNQA (**5**, EC_50_ = 226.5 µg/mL) showed the least DPPH radical scavenging activity. The DPPH titration curves for fractions **FrA**-**G** (Figure S8) and compounds **1** and **6** (Figure S9) are provided in the Supplementary Materials.

### 3.3. Cytotoxicity of S. incisifolium Fractions Against HT-29 and Caco-2 Cancer Cells

Cytotoxicity towards HT-29 and Caco-2 colorectal cancer cell lines was evaluated for *S. incisifolium* extracts. The most cytotoxic *S. incisifolium* fractions yielded IC_50_ values < 50 µg/mL on HT-29 and Caco-2 cells (Table 2). This is consistent with studies that have been done to show the selectivity of *Sargassum* extracts towards HT-29 and Caco-2 cell apoptosis [21]. **Fr F** (fucoxanthin, **3**) was the most potent sample tested. **Fr D**, (SHQA, **1**) was found to be more selective towards Caco-2 than HT-29 cells (SI = 1.97). Taken together these results indicate the presence of multiple cytotoxic compounds in the *S. incisifolium* extract. Dose-response curves showing the inhibition of HeLa (Figure S10), HT-29 (Figure S11) and Caco-2 (Figure S12) cell proliferation by **FrA**-**I** are provided in the Supplementary Materials.

### 3.4. PPAR-γ Agonist Activity of SHQA (**1**) and Derivatives

**Figure 3:**
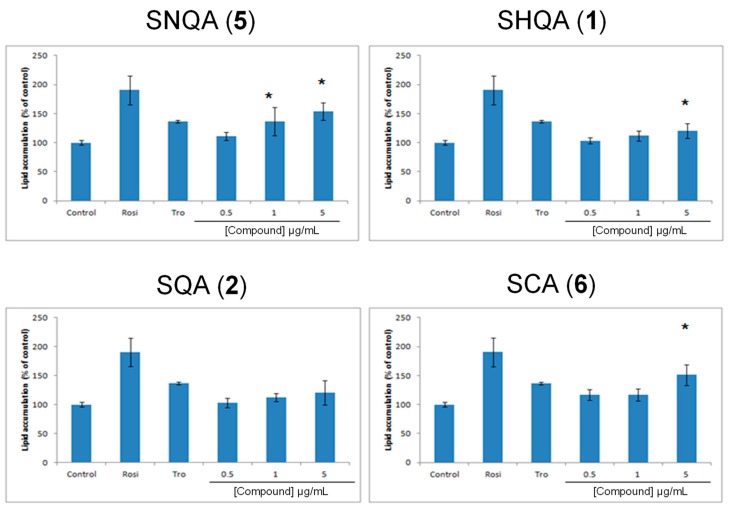
Dose-dependent lipid accumulation in differentiating 3T3-L1 cells after treatment with sargahydroquinoic acid (**1**), sargaquinoic acid (**2**), sarganaphthoquinoic acid (**5**), and sargachromenoic acid (**6**). A total of 1 µM each of rosiglitazone (Rosi) and troglitazone (Tro) was used as positive control. Each data point represents the mean ± SD (n = 3) while the asterisks (*) indicate a significant increase in lipid accumulation.

**Figure 4:**
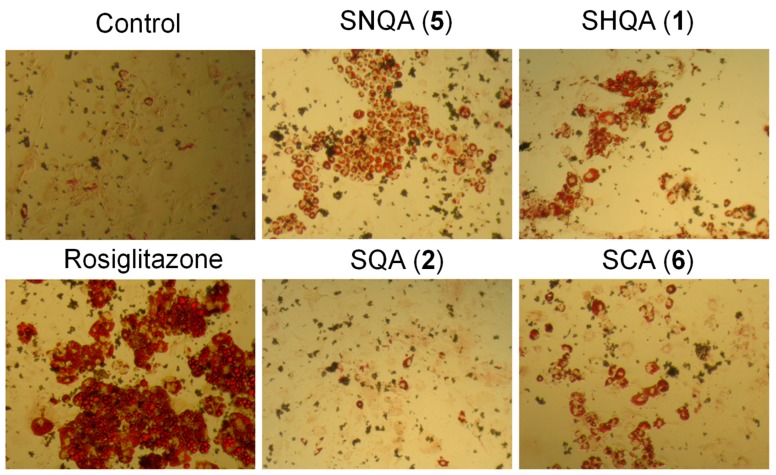
Representative images of oil red O stained 3T3-L1 cells after treatment with sargahydroquinoic acid (**1**), sargaquinoic acid (**2**), sarganaphthoquinoic acid (**5**), sargachromenoic acid (**6**) and rosiglitazone. Original magnification 200×.

Adipocyte differentiation can be used as a convenient indicator of PPAR-γ activation, due to its essential role in adipogenesis [22]. The most significant PPAR-γ response was observed with SNQA (**5**). At test concentrations of 1 µg/mL (0.24 µM) and 5 µg/mL (1.19 µM), SNQA (**5**) produced a response similar to that of rosiglitazone at 1 µM (Figure 3 and Figure 4). SCA (**6**) also induced a strong PPAR-γ response, but it was only significant at 5 µg/mL (1.14 µM). The PPAR-γ activity of SHQA (**1**) was weakly positive at 5 µg/mL (1.17 µM) while SQA (**2**) did not reveal any PPAR-γ activity at all test concentrations. To our knowledge, this is the first report on the PPAR-γ activity of SNQA (**5**).

Fucoxanthin (**3**) and sargaquinal (**4**) showed the strongest cytotoxic activity against HeLa cells with IC_50_ values of 12.11 and 13.59 µg/mL respectively (Table 3). SHQA (**1**) (IC_50_ = 43.16 µg/mL) showed similar activity compared to SNQA (**5**) (IC_50_ = 43.50 µg/mL) and SCA (**6**) (IC_50_ = 53.56 µg/mL), but stronger than its quinone congener, SQA (**2**) (IC_50_ = 92.85 µg/mL). Dose-response curves for the inhibition of HeLa derivative cells (Figure S13) are provided in the Supplementary Materials.

## 4. Discussion

Phytochemical analysis of the major fractions obtained from *S. incisifolium* dichloromethane extract identified SHQA (**1**), SQA (**2**), fucoxanthin (**3**), and sargaquinal (**4**) as the most prominent metabolites present. These represent common constituents often reported to occur in *Sargassum* species. While the biological properties of fucoxanthin (**3**) are plentifully described in the literature, SHQA (**1**), SQA (**2**), and sargaquinal (**4**) remain poorly explored.

Considering that inflammation represents the predominant therapeutic target against IBD [3], the anti-inflammatory potential of the fractions was investigated using LPS activated RAW 264.7 macrophages as a model. Both SHQA (**1**) and fucoxanthin (**3**) revealed a concentration-dependent inhibition in nitrate production, indicating potential anti-inflammatory activity. Concurrent cell viability measurements confirmed that these effects were not due to cytotoxicity. The anti-inflammatory activity of fucoxanthin (**3**) is in accord with previous studies [20]. Similarly, research on the anti-inflammatory activity of plastoquinone derivatives isolated from natural sources also reported that SHQA (**1**) significantly reduced TPA-induced mouse ear oedema [23]. The precise mechanism through which SHQA (**1**) exerts this anti-inflammatory response however awaits further studies. Our results suggest that SHQA (**1**) may, at least in part, directly target macrophage function. In contrast to the anti-inflammatory activity, treatment of naïve RAW 264.7 cells did not reveal any potential risk to induce a pro-inflammatory effect, which could exacerbate inflammation.

Oxidative stress is currently considered as a contributory factor in the initiation, progression, and severity of IBD and is regarded as more than just a simple consequence of chronic inflammation associated with the disease. Although the underlying mechanisms are yet to be thoroughly elucidated, strategies to reduce oxidative stress are anticipated to improve therapeutic outcome. Antioxidants, especially those derived from natural products, have attracted attention as acceptable ingredients to target oxidative stress in IBD [4]. Compounds with dual anti-inflammatory and antioxidant activity may be particularly relevant to the treatment of IBD. The DPPH radical scavenging activity of SHQA (**1**) and SCA (**6**) has been previously documented [23]. Reduced forms of vitamin E and coenzyme Q groups, such as hydroquinones, chromanols, and chromenols normally function as protective anti-oxidants. However, the ability of such compounds to function in these capacities of electron transfer and antioxidant activity directly depend upon the oxidation potential of the compound, which is also partly dependent upon the nuclear substituents [24]. Not surprisingly, SHQA (**1**) showed more potent DPPH radical scavenging activity than SQA (**2**, EC_50_ = 95.76 µg/mL), as it is generally known that hydroquinones are more potent radical scavengers than their quinone congeners. We, therefore, identify SHQA (**1**) as an anti-inflammatory compound with potent radical scavenging activity. In the DPPH assay, SHQA (**1**) was greater than 5-fold more active relative to the standard antioxidant, ascorbic acid.

It is well recognised that patients with IBD show a higher incidence of developing colon cancer [4], primarily believed to be the result of chronic intestinal inflammation. Subsequently, many IBD patients also develop the requirement for cancer therapy, which is accompanied by unique challenges associated with this comorbidity. Often chemotherapeutic drugs damage the intestine, resulting in the remission of IBD. It thus follows that although cytotoxicity towards cancer cells may have an advantage in cancer treatment, it is also at risk of aggravating intestinal inflammation and thus, IBD. Evaluation of the fractions obtained from *S. incisifolium* revealed significant toxicity towards colon cancer cell lines Caco-2 and HT-29 with fucoxanthin (**3**) being the most potent. This is consistent with previous studies which report fucoxanthin (**3**) to inhibit the proliferation of HT-29 and Caco-2 cells through inducing cell cycle arrest in the Go/G1 phase at low concentrations (25 μM) and apoptosis at higher concentrations [25]. SHQA (**1**) was also significantly cytotoxic towards these colon cancer cells.

The adipocyte has been described as “a dynamic cell that plays a fundamental role in energy balance and overall body homeostasis” [26]. The formation of adipocytes (adipogenesis) is a differentiation process governed by transcriptional cascades involving a regulated set of gene expression events [22]. The peroxisome proliferator-activated receptor gamma (PPAR-γ) has been termed as the ‘master regulator’ of adipogenesis sufficient to differentiate fibroblasts into mature adipocytes [27]. The PPAR-γ is therefore not only crucial for adipogenesis but is also a requirement for the maintenance of the differentiated state. Hence, a compound or extract that stimulates the differentiation of preadipocytes into mature adipocytes is considered as a PPAR-γ agonist. The PPAR-γ is predominantly expressed in adipose tissue with lower levels of expression in other tissues, such as cardiac, renal, and hepatic tissues [28]. The association of PPAR-γ activation and consequent adipogenesis with an increase in tissue insulin sensitivity provided the basis for the development of thiazolidinediones as a class of anti-diabetic drugs. Other known PPAR isoforms include α and β/δ, for which dual and pan agonists have been identified.

The high relative expression of PPAR-γ in the colon has stimulated many studies on the role of PPAR-γ in gut health. While early studies focused heavily on the involvement of PPAR-γ in the process of colonic tumor suppression, more recently, research has expanded to include intestinal inflammation and fibrosis, major factors in the pathogenesis of IBD. Identification of the direct involvement of PPAR-γ in the mechanism of action of mesalazine, a clinically effective drug often used to treat ulcerative colitis, has highlighted the anti-inflammatory role of PPAR-γ and renewed the search for novel PPAR-γ agonists to treat IBD [9]. Fibrosis, excessive deposition of extracellular matrix components including collagen, is a common complication of IBD, leading to obstruction and loss of function of the intestine. PPAR-γ agonists can diminish fibrogenesis through the antagonist effects on TGF signalling. Given that the anti-inflammatory drugs currently used to treat IBD are unable to attenuate intestinal fibrosis, new therapeutic approaches are sought with PPAR-γ agonists holding considerable promise. Taken together, it is clear that PPAR-γ has again emerged as an important therapeutic target for the development of new drugs to treat IBD.

Previously Kim et al. demonstrated that SHQA (**1**) and SQA (**2**) could activate PPAR-γ [29]. However, under our experimental conditions, only SHQA (**1**) revealed a statistically significant enhancement in 3T3-L1 differentiation, a marker for PPAR-γ agonist activity. Considering that Kim et al. [29] used 10 µM each of SHQA (**1**) and SQA (**2**) for the induction of differentiation while we used a maximum of 1.17 µM of SHQA (**1**) and 1.18 µM of SQA (**2**), it is highly possible that increasing the test concentrations would also result in an increased PPAR-γ activity of these compounds. To further explore SHQA (**1**) as a potential chemical scaffold in the development of new PPAR-γ agonists, we synthesized derivatives of SHQA (**1**), namely sargaquinoic acid (SQA, **2**), sarganaphthoquinoic acid (SNQA, **5**), and sargachromenoic acid (SCA, **6**). The most significant response was obtained from SNQA (**5**), which at test concentrations of 1 µg/mL (0.24 µM) and 5 µg/mL (1.19 µM), produced a response similar to that of rosiglitazone at 1 µM. SCA (**6**) also induced a PPAR-γ response, but it was only significant at 5 µg/mL (1.14 µM).

The structural derivatives were also evaluated for antioxidant activity and cytotoxicity against HeLa derivative cells. SNQA (**5**) showed a dramatic decrease in radical scavenging activity while SCA (**6**) antioxidant activity remained essentially unchanged. Cytotoxicity towards HeLa derivative cells, a cell line previously shown to be devoid of PPAR-γ protein [30], was unchanged relative to SHQA (**1**).

To our knowledge, this is the first report on the PPAR-γ-mediated activity of SNQA (**5**). In an attempt to improve the side effect profiles of current PPAR-γ agonists, research has explored the replacement of the thiazolidine ring with other ‘acidic head groups’, which have lesser side effects. Such examples include a study performed by Sundriyal et al. in which, after replacement of the thiazolidine ring with a 1,4-naphthoquinone moiety, the newly synthesized compounds still retained PPAR-γ activity comparable to pioglitazone [31]. This shows that the 1,4-naphthoquinone, SNQA (**5**), is a potential PPAR-γ agonist similar to the well-known thiazolidinediones and supports our findings and potential for the treatment of IBD. Additionally, 1,4-naphthoquinones are commercially available, less costly, and easily derivatized.

## 5. Conclusions

SHQA (**1**) is identified as a promising lead compound due to its effects on multiple therapeutic targets relevant to IBD. Derivatization to SNQA (**5**) significantly improved the PPAR-activity. However, this dramatically reduced its antioxidant activity and had minimal effect on cytotoxicity.

## Figures and Tables

**Table 1:** DPPH radical scavenging activity of *S. incisifolium* fractions, compounds and analogs.

Fraction/Compound	EC_50_ (µg/mL)
Ascorbic Acid	24.7
Fr A	>500
Fr B	113.9
Fr C	19.48
Fr D (SHQA, **1**)	4.01
Fr E	3.32
Fr F (fucoxanthin, **3**)	N.D ^1^
Fr G	43.14
Sargaquinal (**4**)	N.D ^1^
SQA (**2**)	95.76
SNQA (**5**)	226.5
SCA (**6**)	6.99

^1^ N.D = Not determined.

**Table 2:** Cytotoxicity of *S. incisifolium* fractions against proliferating HT-29 and Caco-2 cells. IC_50_ values as determined using MTT viability assay after 48 h of treatment.

Fraction/Compound	IC_50_ (µg/mL)		Selectivity Index *
	HT-29	Caco-2	
Fr A	150.2	126.8	1.2
Fr B	25.9	14.06	1.8
Fr C	35.06	23.12	1.5
Fr D (SHQA, **1**)	114.8	58.25	1.97
Fr E	369.5	114.1	3.2
Fr F (fucoxanthin, **3**)	19.83	14.82	1.3
Fr G	116.5	117.6	1.0
Fr I	Non-toxic	Non-toxic	-

* Selectivity index: IC_50_ (HT-29)/IC_50_ (Caco-2).

**Table 3:** Cytotoxicity assay results for compounds **1**–**6** against HeLa derivative cells.

Compound	HeLa IC_50_ (µg/mL)
SHQA (**1**)	43.16
SQA (**2**)	92.85
Fucoxanthin (**3**)	12.11
Sargaquinal (**4**)	13.59
SNQA (**5**)	43.50
SCA (**6**)	53.56

## Supplementary Materials

The following are available online at https://www.mdpi.com/2305-6320/6/2/49/s1, Figure S1: Photograph of *S. incisifolium* specimen used in this study, Figure S2: ^1^H NMR spectra (CDCl_3_, 400 MHz) for sargahydroquinoic acid (**1**), sargaquinoic acid (**2**), sargachromenoic acid (**6**) and sarganaphthoquinoic acid (**5**), Figure S3: ^13^C NMR spectrum (CDCl_3_, 100 MHz) of sargahydroquinoic acid (**1**), Figure S4: ^1^H NMR spectrum (CDCl_3_, 400 MHz) of 10′*E*-sargaquinal (**4**), Figure S5: ^13^C NMR spectrum (CDCl_3_, 100 MHz) of 10′*E*-sargaquinal (**4**), Figure S6: ^1^H NMR spectrum (CDCl_3_, 400 MHz) of fucoxanthin (**3**), Figure S7: ^13^C NMR spectrum (CDCl_3_, 100 MHz) of fucoxanthin (**3**), Figure S8: Titration curves for the DPPH radical scavenging activity of *Sargassum incisifolium* crude fractions **Fr A**, **Fr B**, **Fr C**, **Fr D**, **Fr E** and **Fr G**, Figure S9: Titration curves for the DPPH radical scavenging activity of SHQA (**1**) and SCA (**6**), Figure S10: Dose-dependent inhibition of HeLa cell viability by *S. incisifolium* fractions **Fr A**–**I**, Figure S11: Dose-dependent inhibition of HT-29 cell viability by *S. incisifolium* fractions, Figure S12: Dose-dependent inhibition of Caco-2 cell viability by *S. incisifolium* fractions, Figure S13: Dose-dependent inhibition of HeLa cell viability by sarganaphthoquinoic acid (**5**), sargahydroquinoic acid (**1**), sargaquinoic acid (**2**) and sargachromenoic acid (**6**), Scheme S1: Isolation of compounds **1**, **3**, and **4** from *S. incisifolium*, Scheme S2: Semi-synthetic derivatization of sargahydroquinoic acid (**1**) analogs; **2**, **5**, and **6**.

## References

[1] Ng S.C., Shi H.Y., Hamidi N., Underwood F.E., Tang W., Benchimol E.I., Panaccione R., Ghosh S., Wu J.C.Y., Chan F.K.L. (2017). Worldwide incidence and prevalence of inflammatory bowel disease in the 21st century: A systematic review of population-based studies. Lancet.

[2] Duijvestein M., Battat R., Vande Casteele N., D’Haens G.R., Sandborn W.J., Khanna R., Jairath V., Feagan B.G. (2018). Novel Therapies and Treatment Strategies for Patients with Inflammatory Bowel Disease. Curr. Treat. Opt. Gastroenterol..

[3] Verstockt B., Ferrante M., Vermeire S., Van Assche G. (2018). New treatment options for inflammatory bowel disease. J. Gasteroenterol..

[4] Tian T., Wang Z., Zhang J. (2017). Pathomechanisms of oxidative stress in inflammatory bowel disease and potential antioxidant therapies. Oxid. Med. Cell. Longev..

[5] Su C.G., Wen X., Bailey S.T., Jiang W., Rangwala S.M., Keilbaugh S.A., Flanigan A., Murthy S., Lazar M.A., Wu G.D. (1999). A novel therapy for colitis utilizing PPAR-γ ligands to inhibit the epithelial inflammatory response. J. Clin. Investig..

[6] Dubuquoy L., Rousseaux C., Thuru X., Peyrin-Biroulet L., Romano O., Chavatte P., Chamaillard M., Desreumaux P. (2006). PPARγ as a new therapeutic target in inflammatory bowel diseases. Gut.

[7] Annese V., Rogai F., Settesoldi A., Bagnoli S. (2012). PPARγ in inflammatory bowel disease. PPAR Res..

[8] Wang X., Sun Y., Zhao Y., Ding Y., Zhang X., Kong L., Li Z., Guo Q., Zhao L. (2016). Oroxyloside prevents dextran sulfate sodium-induced experimental colitis in mice by inhibiting NF-ķB pathway through PPARγ activation. Biochem. Pharmacol..

[9] Schwab M., Reynders V., Loitsch S., Shastri Y.M., Steinhilber D., Schröder O., Stein J. (2008). PPARγ is involved in mesalazine-mediated induction of apoptosis and inhibition of cell growth in colon cancer cells. Carcinogenesis.

[10] Stolfi C., Pallone F., Monteleone G. (2012). Colorectal cancer chemoprevention by mesalazine and its derivatives. J. Biomed. Biotech..

[11] Harvey A., Edrada-Ebel R., Quinn R.J. (2015). The re-emergence of natural products for drug discovery in the genomics era. Nat. Rev. Drug Discov..

[12] Dias D.A., Urban S., Roessner U. (2012). A historical overview of natural products in drug discovery. Metabolites.

[13] Poornima P., Kumar J.D., Zhao Q., Blunder M., Efferth T. (2016). Network pharmacology of cancer: From understanding of complex interactomes to the design of multi-target specific therapeutics from nature. Pharmacol. Res..

[14] Mattio L., Anderson R.J., Bolton J.J. (2015). A revision of the genus Sargassum (Fucales, Phaeophyceae) in South Africa. S. Afr. J. Bot..

[15] Mmola M., Le Roes-Hill M., Durrell K., Bolton J.J., Sibuyi N., Meyer M.E., Beukes D.R., Antunes E. (2016). Enhanced antimicrobial and anticancer activity of silver and gold nanoparticles synthesised using *Sargassum incisifolium* aqueous extracts. Molecules.

[16] Afolayan F., Bolton J., Lategan A., Smith P., Beukes D. (2008). Fucoxanthin, Tetraprenylated Toluquinone and Toluhydroquinone Metabolites from Sargassum heterophyllum Inhibit the in vitro Growth of the Malaria Parasite Plasmodium falciparum. Z. Naturforsch..

[17] Kusumi T., Shibata Y., Ishitsuka M., Kinoshita T., Kakisawa H. (1979). Structures of new plastoquinones from the brown alga Sargassum serratifolium. Chem. Lett..

[18] Mori K., Ooi T., Hiraoka M., Oka N., Hamada H., Tamura M., Kusumi T. (2004). Fucoxanthin and Its Metabolites in Edible Brown Algae Cultivated in Deep Seawater. Mar. Drugs.

[19] Munedzimwe T.C., van Zyl R.L., Heslop D.C., Edkins A.L., Beukes D.R. (2019). Semi-synthesis and evaluation of sargahydroquinoic acid derivatives as potential antimalarial agents. Medicines.

[20] Peng J., Yuan J., Wu C., Wang J. (2011). Fucoxanthin, a marine carotenoid present in brown seaweeds and diatoms: Metabolism and bioactivities relevant to human health. Mar. Drugs.

[21] Khanavi M., Nabavi M., Sadati N., Ardekani M.S., Sohrabipour J., Nabavi S.M., Ghaeli P., Ostad S.N. (2010). Cytotoxic activity of some marine brown algae against cancer cell lines. Biol. Res..

[22] Moseti D., Regassa A., Kim W.K. (2016). Molecular regulation of adipogenesis and potential anti-adipogenic bioactive molecules. Int. J. Mol. Sci..

[23] Pérez-Castorena A.L., Arciniegas A., Apan M.T., Villaseñor J.L., de Vivar A.R. (2002). Evaluation of the anti-inflammatory and antioxidant activities of the plastoquinone derivatives isolated from *Roldana barba-johannis*. Planta Medica.

[24] Moore H.W., Schwab D.E., Folkers K. (1964). Coenzyme, Q. LVII. Synthesis of new analogs of coenzyme Q4 for biochemical mechanism studies. Biochemistry.

[25] Das S.K., Hashimoto T., Shimizu K., Yoshida T., Sakai T., Sowa Y., Komoto A., Kanazawa K. (2005). Fucoxanthin induces cell cycle arrest at G0/G1 phase in human colon carcinoma cells through up-regulation of p21WAF1/Cip1. Biochim. Biophys. Acta (BBA)—Gen. Subj..

[26] Bernlohr D.A., Jenkins A.E., Benaars A.A., Vance D.E., Vance J.E. (2002). Adipose tissue and lipid metabolism. Biochemistry of Lipids, Lipoproteins and Membranes.

[27] Ma X., Wang D., Zhao W., Xu L. (2018). Deciphering the Roles of PPARγ in Adipocytes via Dynamic Change of Transcription Complex. Front. Endocrinol..

[28] Janani C., Ranjitha K.B.D. (2015). PPAR gamma gene: A review. Diabetes Metab. Syndr. Clin. Res. Rev..

[29] Kim S.N., Choi H.Y., Lee W., Park G.M., Shin W.S., Kim Y.K. (2008). Sargaquinoic acid and sargahydroquinoic acid from *Sargassum yezoense* stimulate adipocyte differentiation through PPARα/γ activation in 3T3-L1 cells. FEBS Lett..

[30] Lin Y., Wang F., Yang L., Chun Z., Bao J., Zhang G. (2013). Anti-inflammatory phenanthrene derivatives from stems of *Dendrobium denneanum*. Phytochemistry.

[31] Sundriyal S., Viswanad B., Bharathy E., Ramarao P., Chakraborti A.K., Bharatam P.V. (2008). New PPARγ ligands based on 2-hydroxy-1,4-naphthoquinone: Computer-aided design, synthesis, and receptor-binding studies. Bioorgan. Med. Chem. Lett..

