# Supplementary material for: In Vitro Evaluation of the Phytopharmacological Potential of Sargassum incisifolium for the Treatment of Inflammatory Bowel Diseases
Source: Medicines (Basel). 2019 Apr 6;6(2):49. doi: 10.3390/medicines6020049 (PMC6631261; doi:10.3390/medicines6020049)
Supplement: Supplementary file 1 [file medicines-06-00049-s001.pdf]

Supplementary Materials: In Vitro Evaluation of the Phytopharmacological Potential of *Sargassum incisifolium* for the Treatment of Inflammatory Bowel Diseases

Mutenta N. Nyambe, Trevor C. Koekemoer, Maryna van de Venter, Eleonora D. Goosen and Denzil R. Beukes

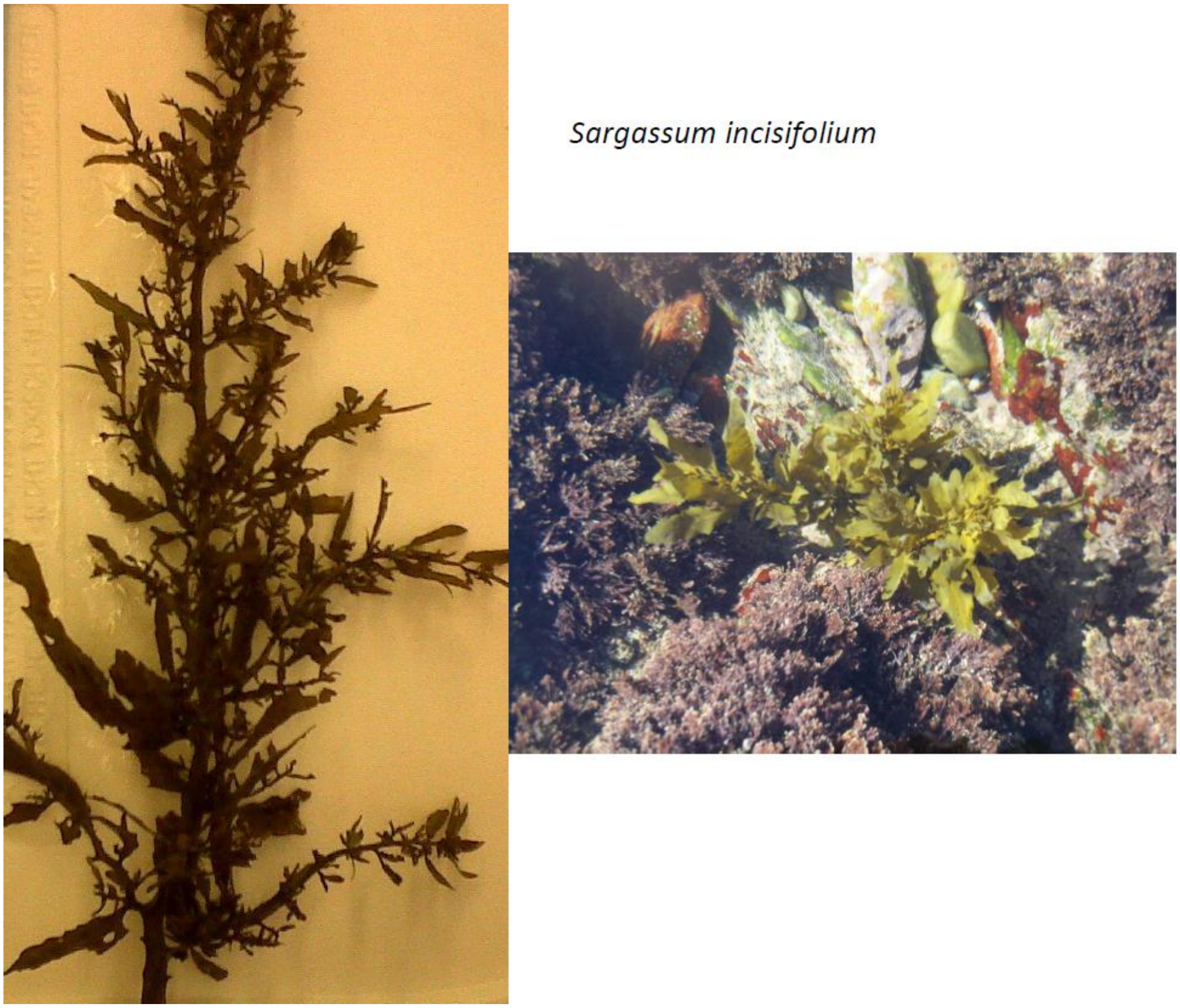

Figure S1. Photograph of *S. incisifolium* specimen used in this study.

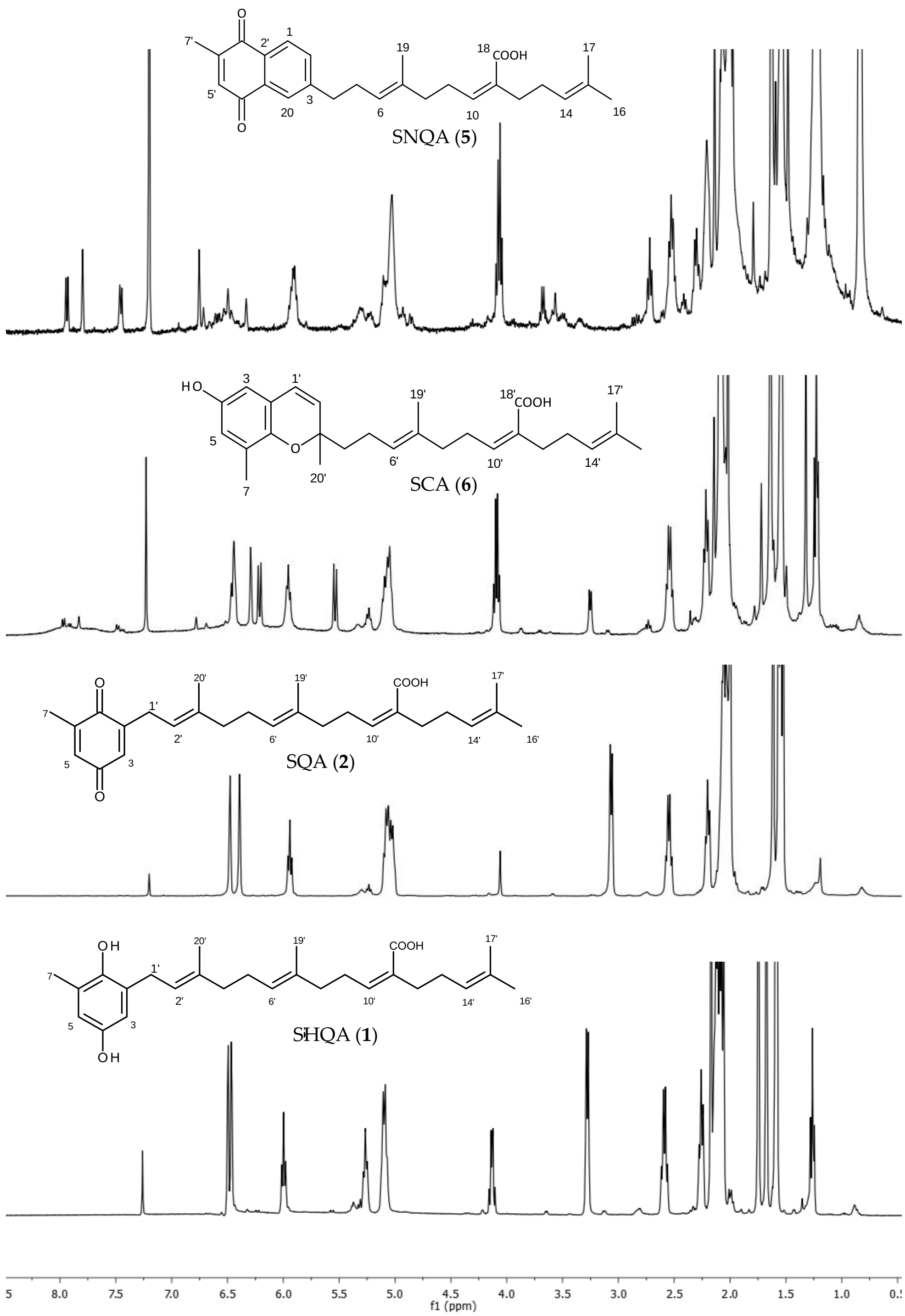

Figure S2. ^{1}H NMR spectra (CDCl3, 400 MHz) for sargahydroquinoic acid (**1**), sargaquinoic acid (**2**), sargachromenoic acid (**6**) and sarganaphthoquinoic acid (**5**).

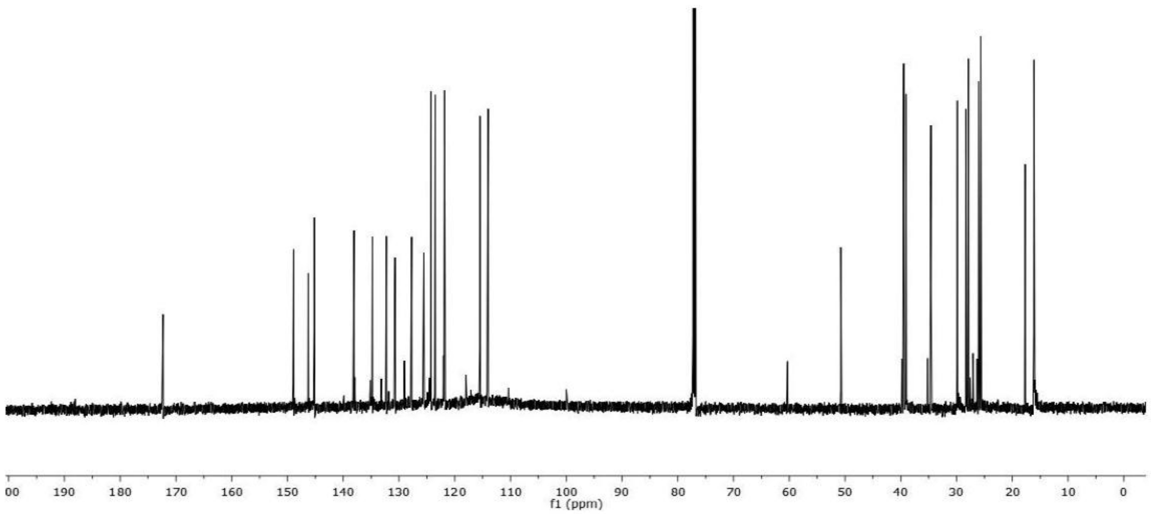

Figure S3. ^{13}C NMR spectrum (CDCl3, 100 MHz) of sargahydroquinoic acid (**1**).

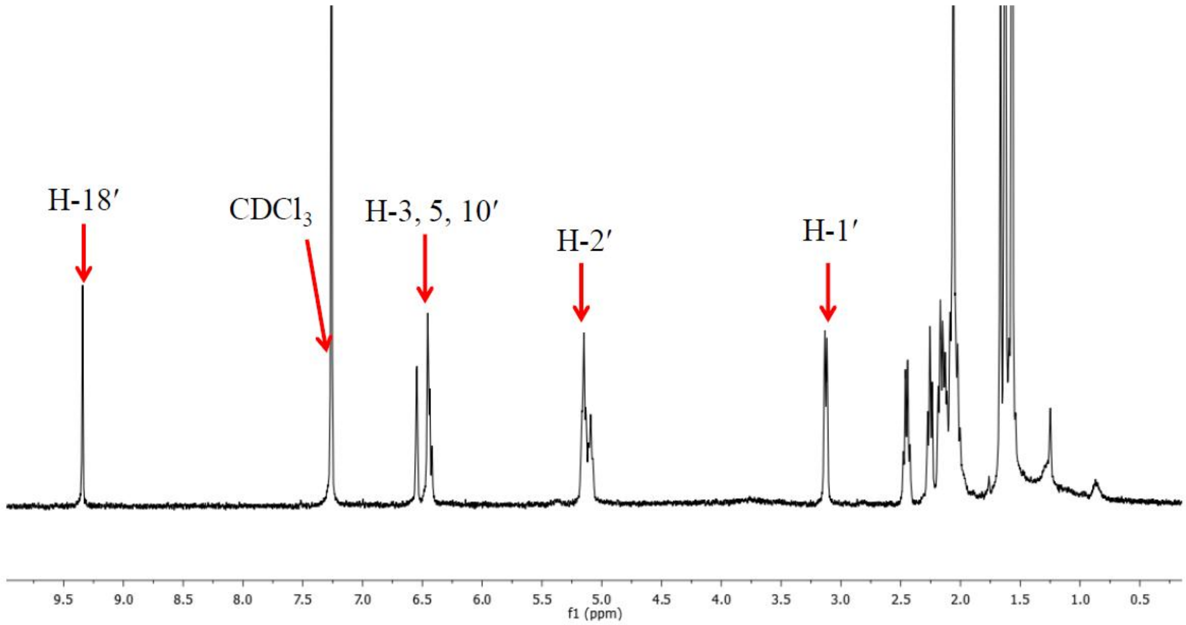

Figure S4. ^{1}H NMR spectrum (CDCl3, 400 MHz) of 10′*E*-sargaquinal (**4**).

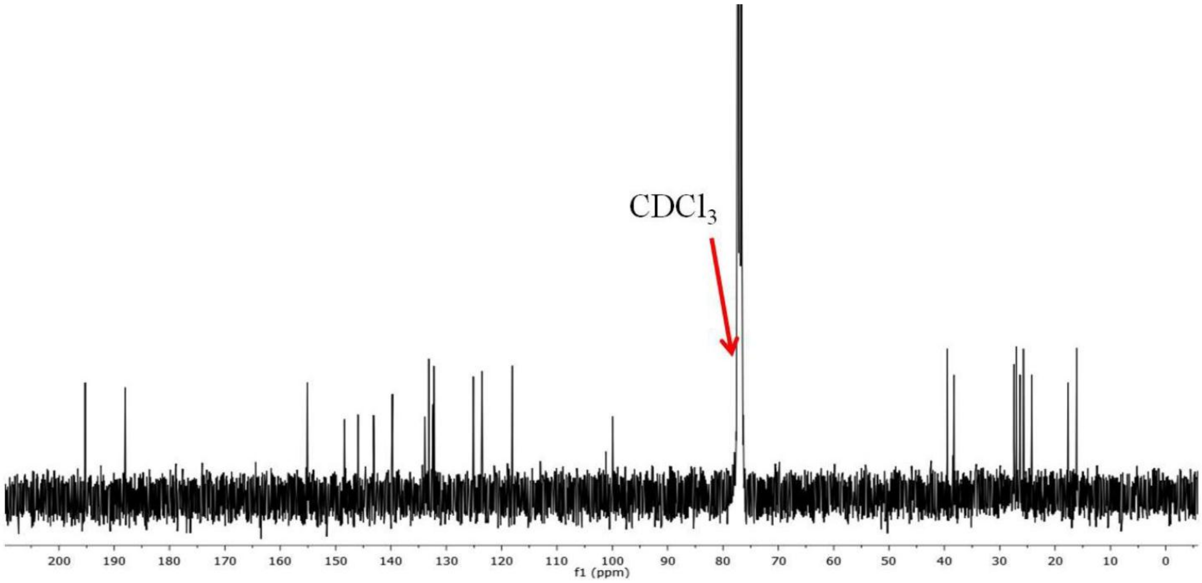

Figure S5. ^{13}C NMR spectrum (CDCl3, 100 MHz) of 10′*E*-sargaquinal (**4**).

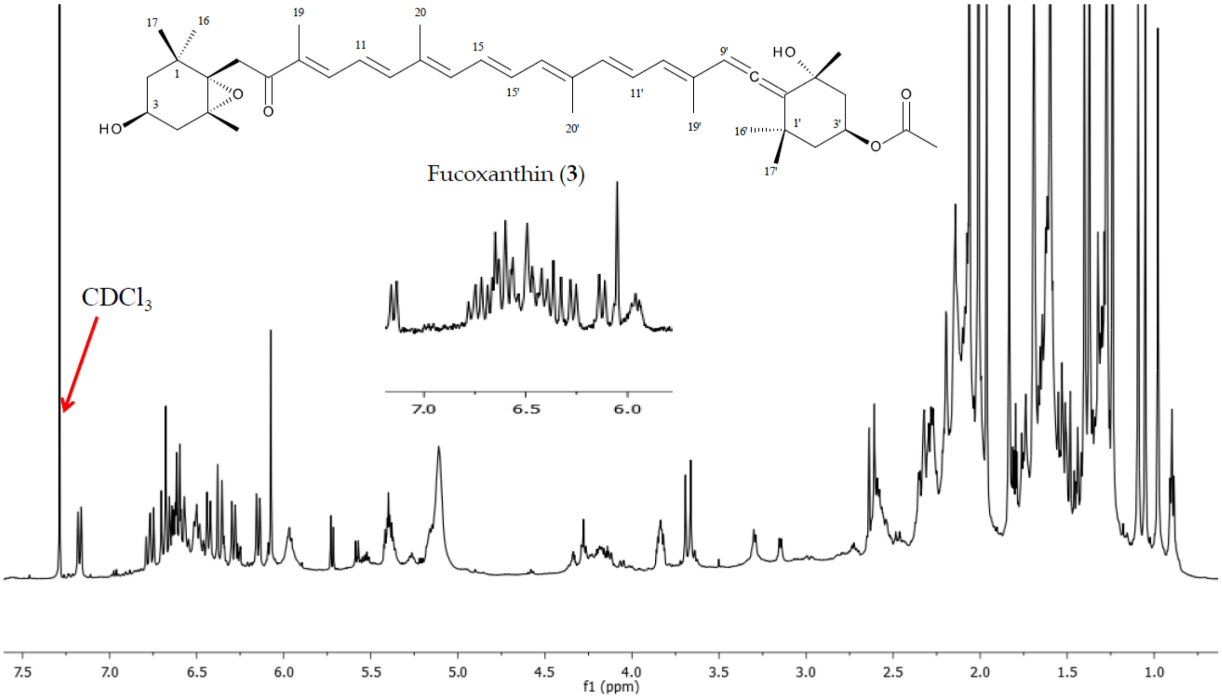

Figure S6. ^{1}H NMR spectrum (CDCl3, 400 MHz) of fucoxanthin (**3**).

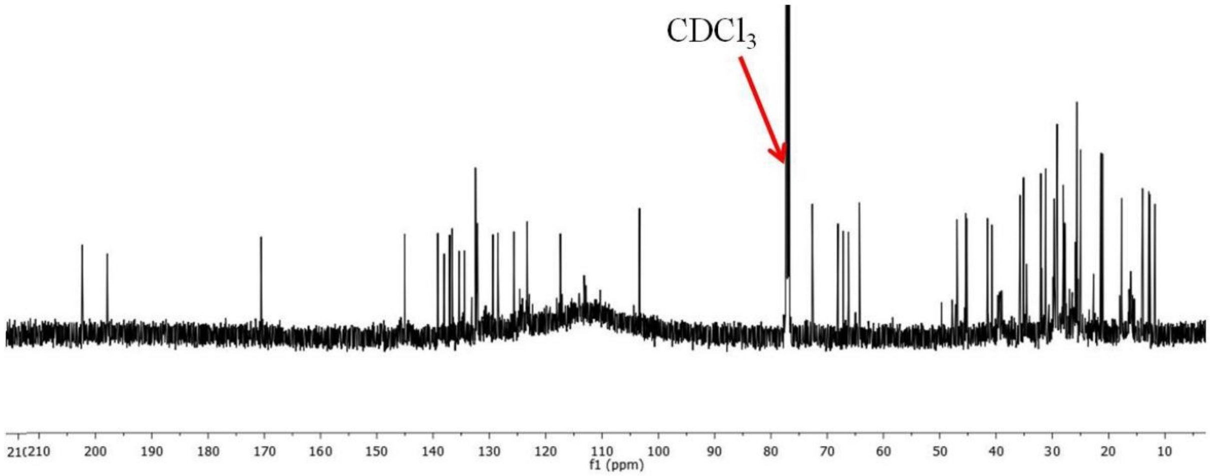

Figure S7. ^{13}C NMR spectrum (CDCl3, 100 MHz) of fucoxanthin (**3**).

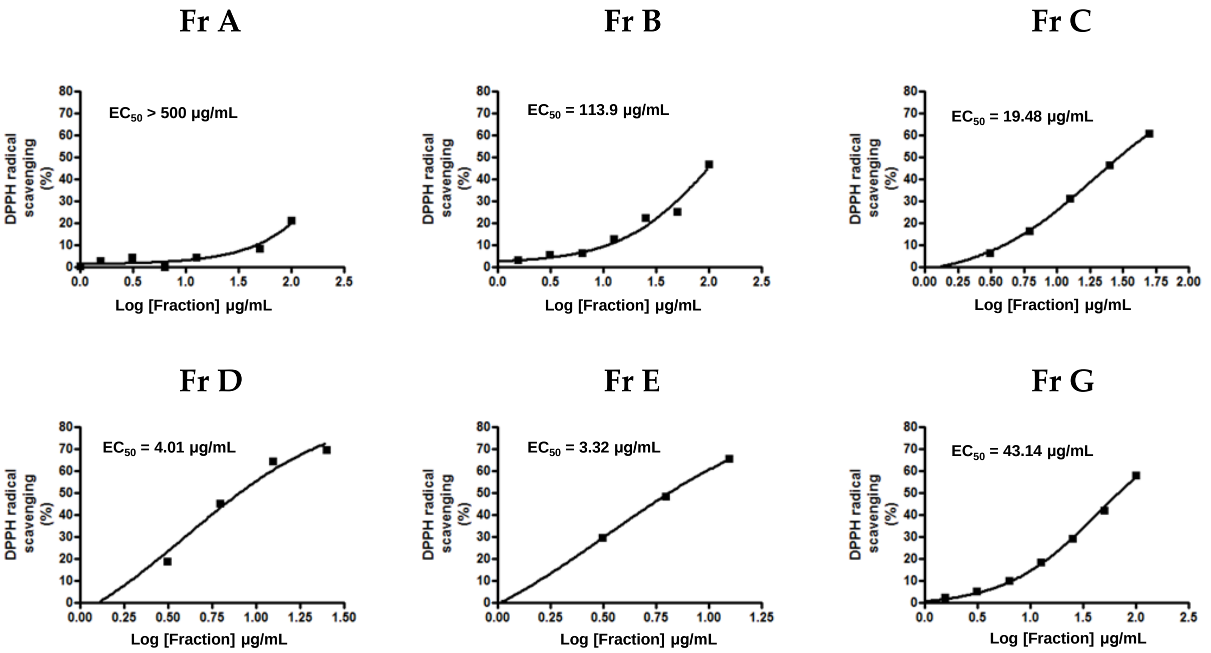

Figure S8. Titration curves for the DPPH radical scavenging activity of *Sargassum incisifolium* crude fractions **Fr A**, **Fr B**, **Fr C**, **Fr D**, **Fr E** and **Fr G**.

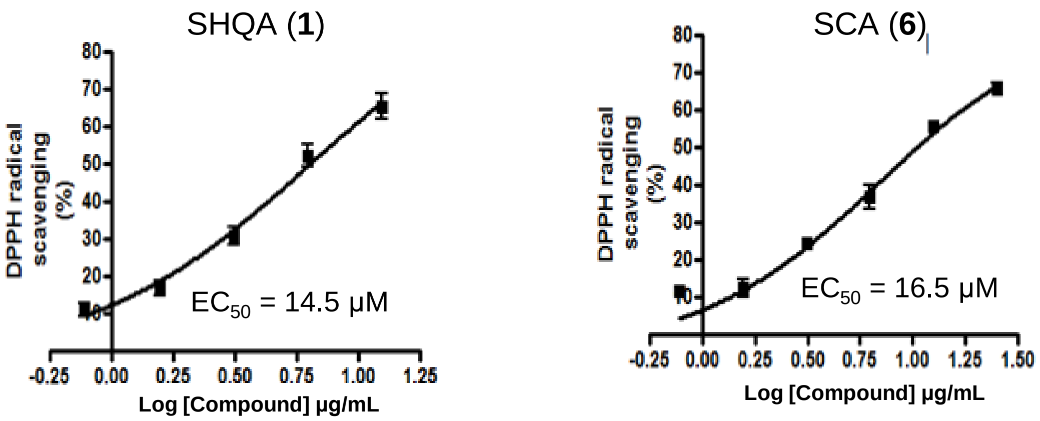

Figure S9. Titration curves for the DPPH radical scavenging activity of SHQA (**1**) and SCA (**6**).

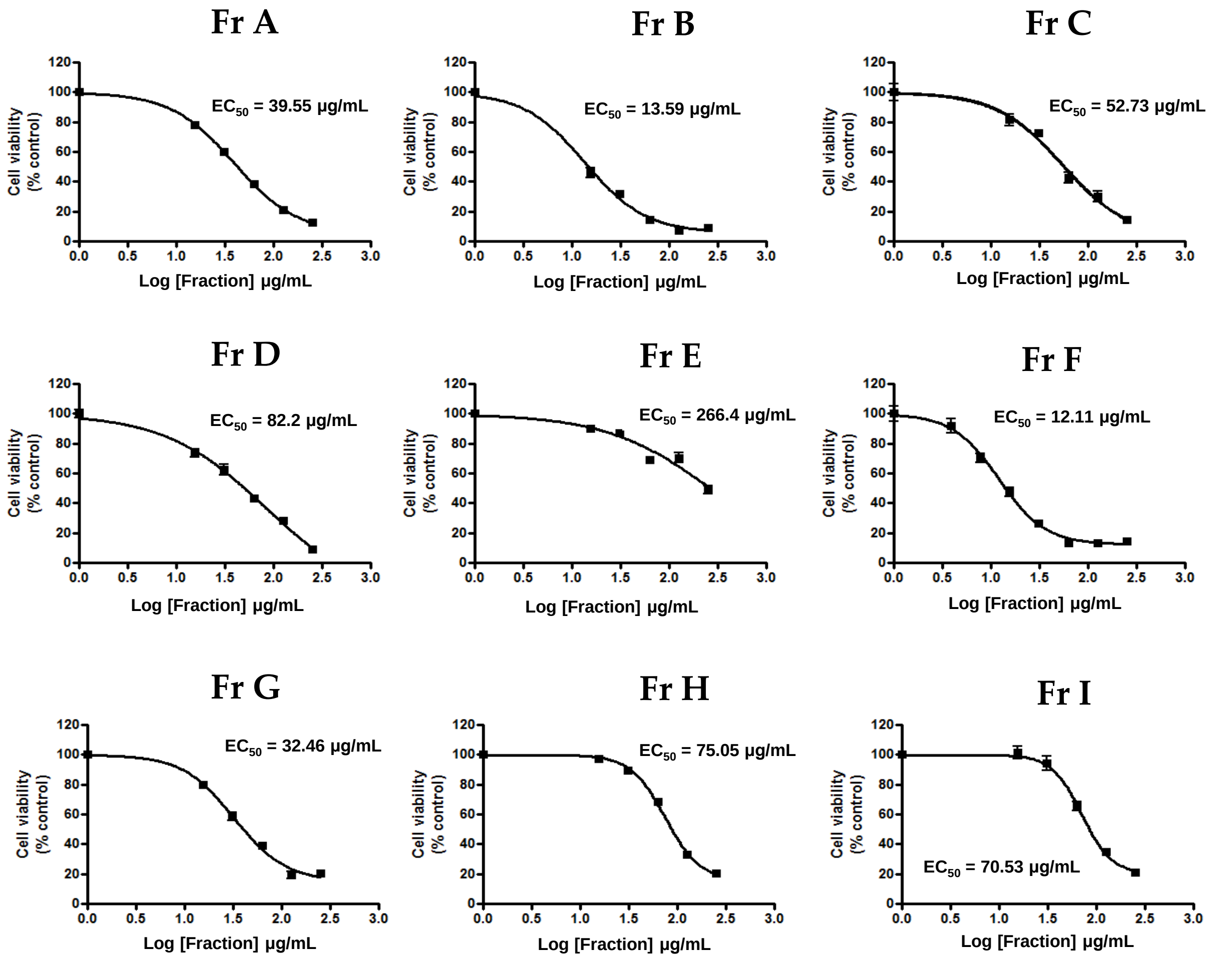

Figure S10. Dose dependent inhibition of HeLa cell viability by *S. incisifolium* fractions **Fr A–I**.

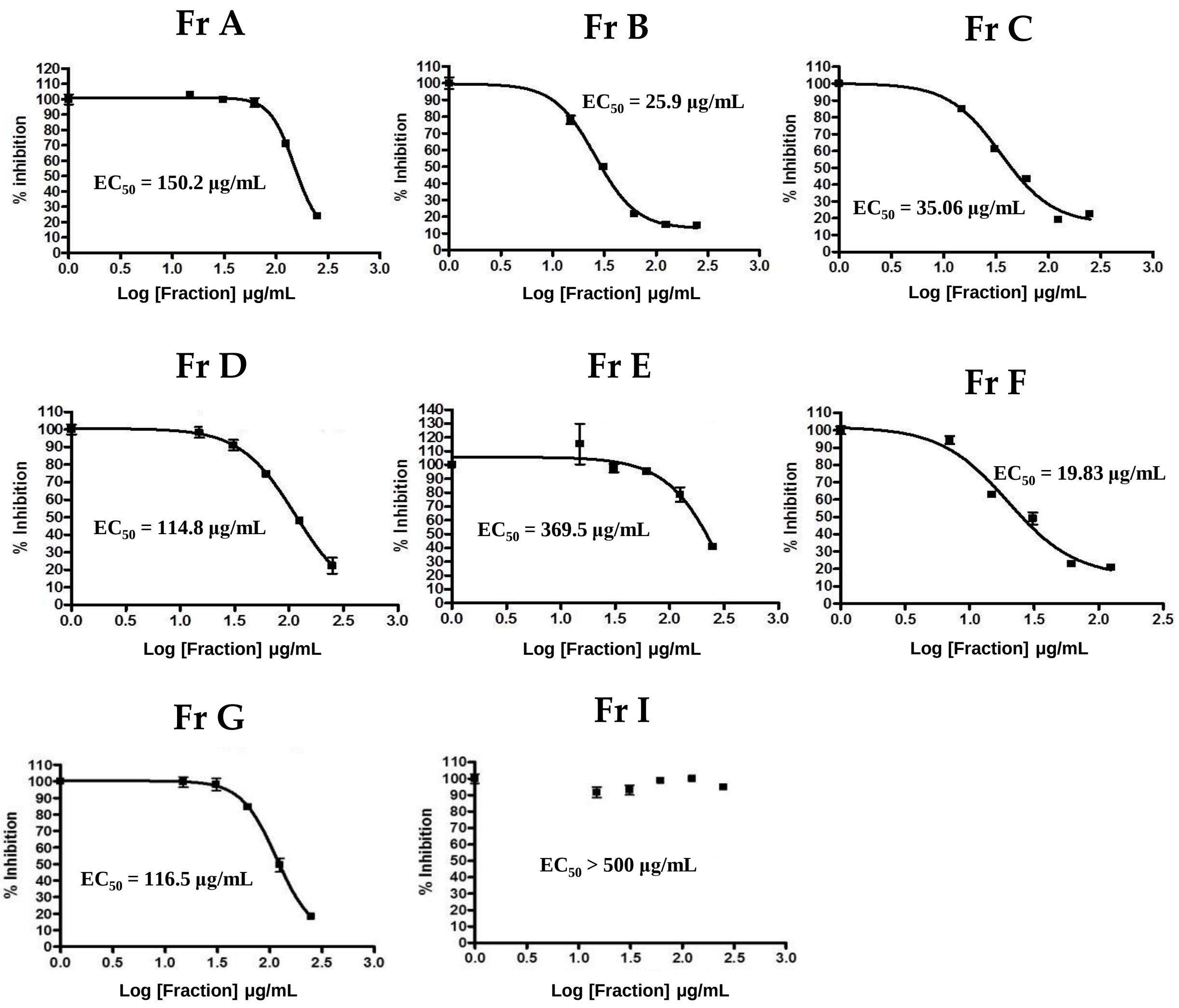

Figure S11. Dose dependent inhibition of HT-29 cell viability by *S. incisifolium* fractions.

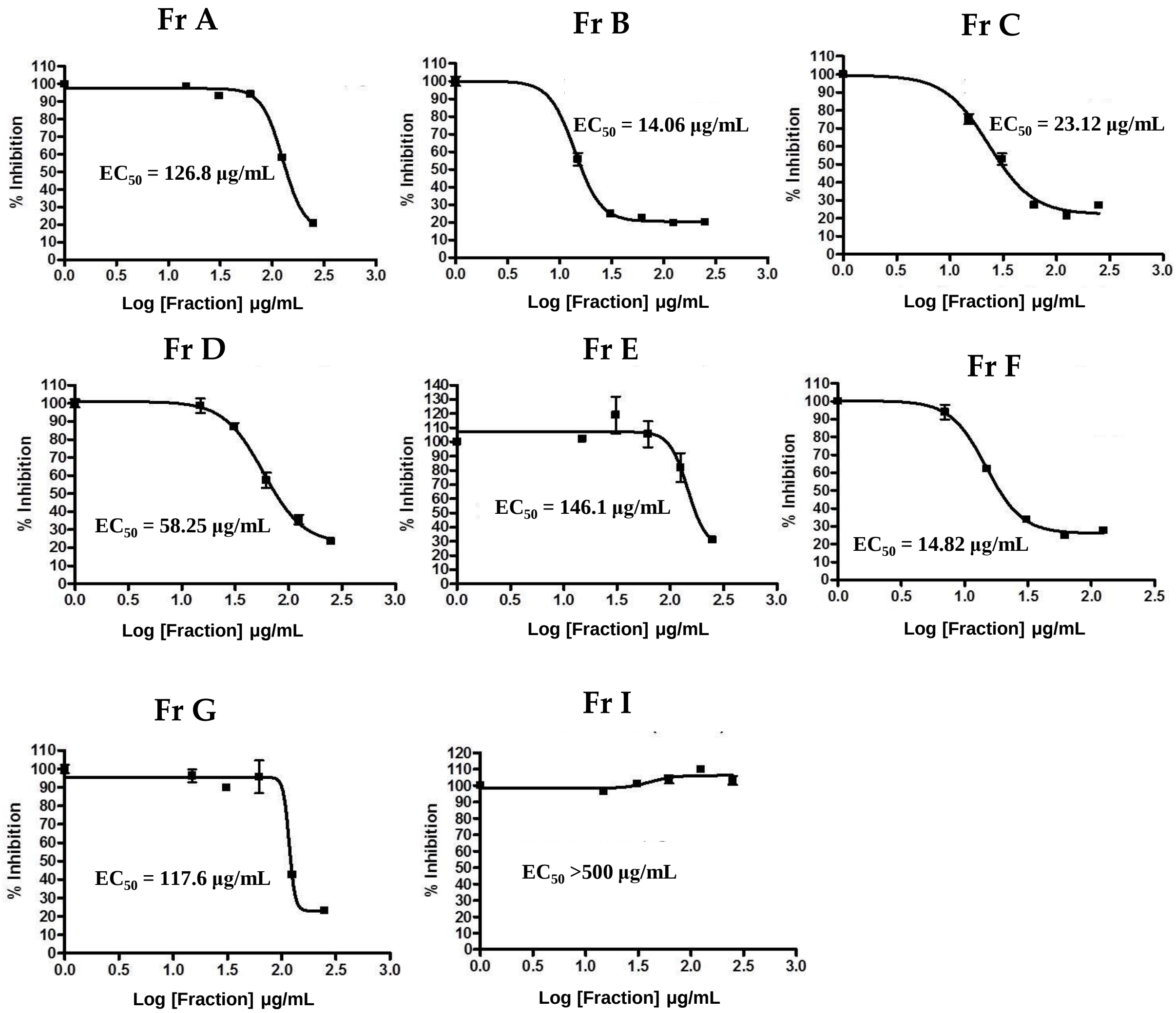

Figure S12. Dose dependent inhibition of Caco-2 cell viability by *S. incisifolium* fractions.

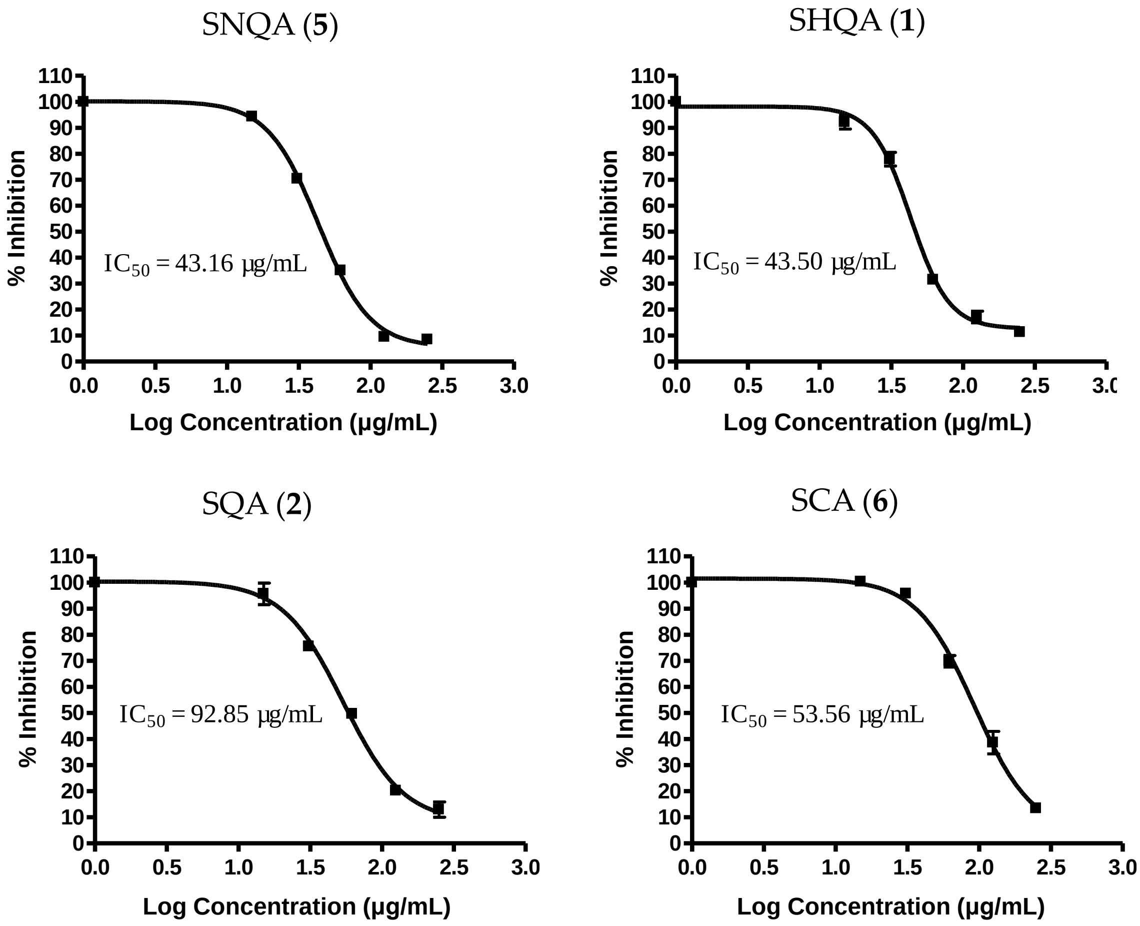

Figure S13. Dose dependent inhibition of HeLa cell viability by sarganaphthoquinoic acid (**5**), sargahydroquinoic acid (**1**), sargaquinoic acid (**2**) and sargachromenoic acid (**6**).

Extraction and isolation

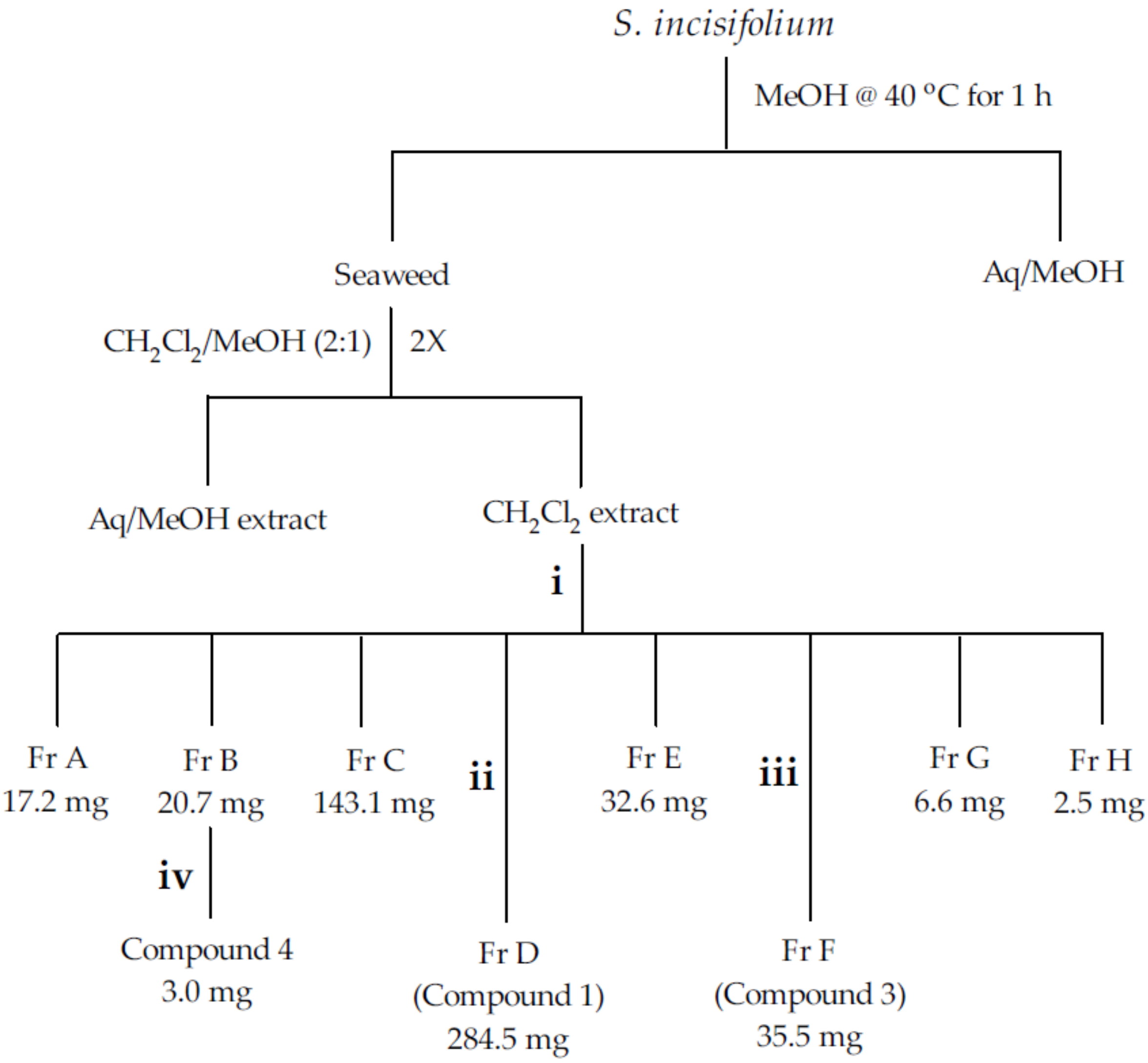

Scheme S1. Isolation of compounds **1**, **3**, and **4** from *S. incisifolium*. Conditions: (**i**) Step gradient silica gel column chromatography, mobile phase *n*-hexane-EtOAc. (**ii**) Silica gel column fraction, *n*-hexane-EtOAc (7:3). (**iii**) Silica gel column fraction, *n*-hexane-EtOAc (4:6). (**iv**) Normal Phase HPLC fraction, *n*-hexane- EtOAc (9:1).

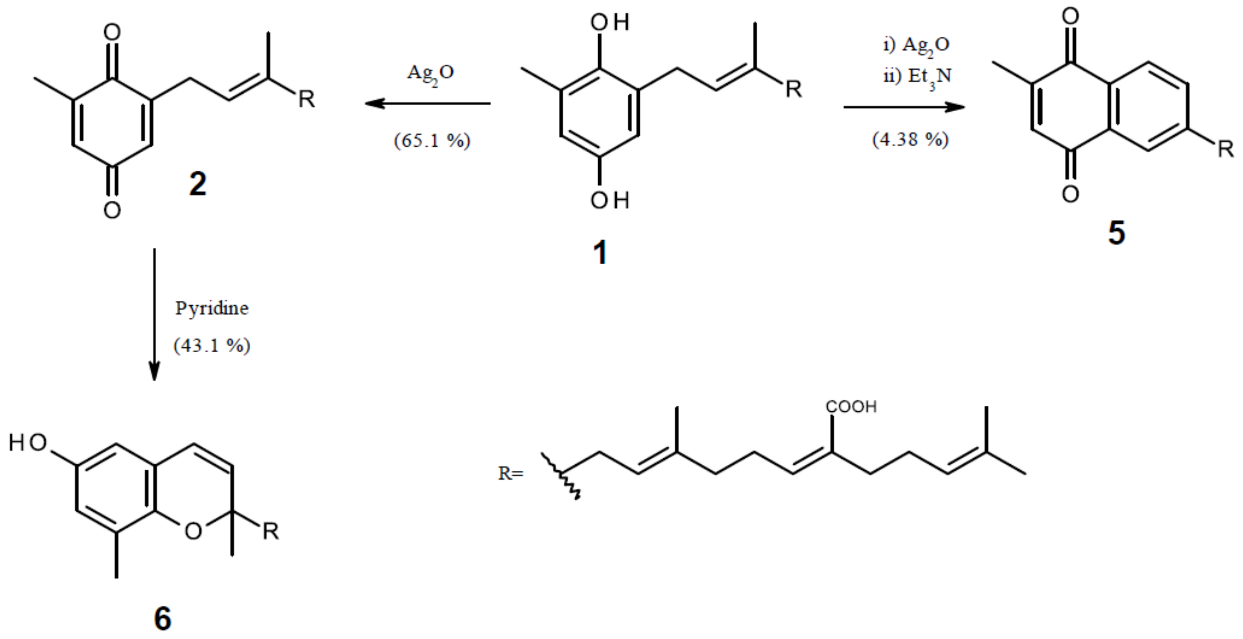

Scheme S2. Semi-synthetic derivatization of sargahydroquinoic acid (**1**) analogs; **2**, **5**, and **6**.
